# Editorial: Acute symptomatic seizures and epileptiform abnormalities: Management and outcomes

**DOI:** 10.3389/fneur.2023.1185710

**Published:** 2023-03-29

**Authors:** Vineet Punia, Marian Galovic, Zhibin Chen, Carla Bentes

**Affiliations:** ^1^Epilepsy Center, Cleveland Clinic, Cleveland, OH, United States; ^2^Department of Neurology, Clinical Neuroscience Center, University Hospital and University of Zürich, Zürich, Switzerland; ^3^Department of Neuroscience, Central Clinical School, Monash University, Melbourne, VIC, Australia; ^4^Department of Medicine – Royal Melbourne Hospital, University of Melbourne, Parkville, VIC, Australia; ^5^Reference Centre for Refractory Epilepsies (Member of EpiCARE), Hospital de Santa Maria-CHULN, Lisbon, Portugal; ^6^Department of Neuroscience and Mental Health (Neurology), Hospital de Santa Maria-CHULN, Lisbon, Portugal; ^7^Centro de Estudos Egas Moniz, Faculdade de Medicina da Universidade de Lisboa, Lisbon, Portugal

**Keywords:** anti-seizure medication (ASM), acute symptomatic seizure, PASS clinic, epileptogenesis, continuous EEG (cEEG)

Every brain can produce an epileptic seizure. A key distinction is whether it is provoked or unprovoked. This dichotomy alludes to the identification of a temporally-associated etiology. When it is present at the time of, or immediately preceding, a seizure, they are called acute symptomatic seizures (ASyS). The insult-to-ASyS time window is etiology-dependent, ranging from 24 h to 7 days and longer (1). Conceptually, the idea of seizure risk reduction after reversing underlying etiology is quite appealing. However, most ASyS are secondary to a non-reversible etiology, such as acute brain injuries (2), and exert a far more significant impact than traditionally appreciated. Assumptions and assertions about these “transitory” events have prevented systematic investigations into ASyS management, outcomes, and its natural history determination. The most glaring example underlies its fundamental defining feature—seizures within 7 days of acute brain injuries—proposed initially for “epidemiological studies” (1), which now pervades clinical practice. While seizures after the arbitrarily chosen 7 days have different outcomes than ASyS (3), emerging data suggest that ASyS after 3 days of injuries like stroke have similar implications (4). Needless to say, it is time to rethink ASySs and their standing in clinical epileptology.

Convulsive ASyS are ubiquitous in clinical practice and, depending on geographical location, account for 40–50% of all afebrile seizures (5). Convulsive ASyS, including ones from metabolic insults, undoubtedly increases the risk of epilepsy development (epileptogenesis) (5, 6). Prognostic models for symptomatic epilepsy development find ASyS contributing the highest risk among predictors of epileptogenesis (7, 8). As a corollary to the classic dictum of *seizure begets seizure* (9), if a brain can generate a seizure (ASyS) once, it is easier for it to produce an unprovoked, remote symptomatic seizure as well, i.e., remote symptomatic epilepsy (10). In other words, ASyS may be a marker of a lower seizure threshold in an individual.

The risk of symptomatic epilepsy after ASyS in stroke patients is 33% (3), precisely similar to the risk of developing epilepsy after a first unprovoked seizure (11). The SeLECT score, a prognostic model for ischemic stroke, predicts more than 60% risk of epilepsy development within a year of ASyS in patients with MCA cortical stroke and more than 3 NIHSS (7), suggesting that epilepsy can be diagnosed at the time of ASyS in some patients (10). These high seizure recurrence risk predictions can have socio-economic ramifications for patients, including driving restrictions.

Convulsive ASyS represents only “*the tip of the iceberg*” when it comes to acute epileptogenic activity after brain injuries. ASyS prevalence is higher in the era of continuous EEG (cEEG) monitoring because most are non-convulsive, i.e., electrographic seizures, during hospitalization (12, 13). In addition, epileptiform abnormalities (EAs) such as lateralized periodic discharges (LPDs), lateralized rhythmic delta activity (LRDA), etc., which significantly increase ASyS risk, are present in 25–40% of patients undergoing acute EEG (14, 15). Like convulsive ASyS, these electrographic findings also increase epilepsy development risk (14, 16–18). Based on this evidence, it is no exaggeration that ASyS and acute EAs may represent the earliest stage of epileptogenesis. Hence, ignoring ASyS and EAs as an epiphenomenon of acute injury is a heavy loss of opportunity for enhancing our understanding of epileptogenesis biomarkers and targets for testing anti-epileptogenic therapies—the holy grail of epilepsy care.

Mortality after ASyS is nine times higher than unprovoked seizures, with a 30-day case fatality of 20% (3, 19). Primary ASyS prophylaxis using anti-seizure medications (ASMs) is recommended after brain injuries, like trauma (20), but not stroke and hemorrhages (21, 22). Some experts recommend ASM prophylaxis after ASyS in intracerebral hemorrhage (ICH) (22) or after “recurrent” ASyS in ischemic stroke (21). In contrast, some organizations recommend against secondary ASM prophylaxis after ischemic stroke (23), and we lack data to support its use after infections (24). Nonetheless, real-world data shows that ASyS and EAs are frequently treated with ASMs during hospitalization, and patients are discharged on them (25–27). While 20% ASyS present as status epilepticus (6), 100% are treated with a status epilepticus management algorithm. The costs and benefits of this treatment strategy for ASyS remain unknown. The *unknowns* abound in this sphere—convulsive vs. electrographic ASyS management, wisdom of prophylactically treating EAs to prevent ASyS, duration of inpatient therapy, and need for discharging patients on ASMs after ASyS—are all unknowns. The latter does not show any benefit in neonates (28). Due to a lack of data guiding optimal ASM duration in adults, a majority continue ASMs several months to years after hospital discharge (29, 30). There is a large variability of expert recommendation on the duration of ASM continuation after ASyS and EAs ranging from months to years (31, 32). In the absence of anti-epileptogenic therapies, there is an acute need for developing evidence-based management strategies in this patient population.

This research collection aims to collate the latest research and review articles concerning ASyS and EAs, their implications, and management. Fatima et al. found that the evolution of LPD's amplitude over time in a patient correlates with seizure risk. Martinez et al. found that nearly a quarter of suspected ASyS patients undergoing cEEG monitoring have the poorly understood phenomenon of stimulus-induced, rhythmic, periodic, or ictal discharges (SIRPIDs), especially common in acute systemic illness, and may correlate with poor outcomes. Pan et al. report that a lower partial pressure of carbon dioxide (PaCO_2_) in intracerebral hemorrhage patients could be associated with an increased risk of hyperacute (<24 h) ASyS. Yu et al. found that late symptomatic seizures (>12 months), rather than ASyS after moderate to severe traumatic brain injury, are associated with unfavorable long-term (5 years) functional outcomes. Tako et al. report that the severity of large arterial vessel occlusion ischemic stroke, based on the NIHSS at 24 h after admission, has a small but significant association with subsequent ASyS. Germeraad et al. explore the age-old question of primary ASyS prophylaxis in the unique setting of hematopoietic stem cell transplantation with busulfan conditioning and report that phenytoin use may cause more harm than benefit and hence recommend against it. Asnakew et al. report that in a specific Ethiopian region and community, illiteracy is the primary driver of people's attitude and care toward people with seizures, ASyS, or otherwise. Sharma et al. provide a comprehensive, concise, and clinically helpful review of the role of cEEG in managing patients with suspected ASyS, including challenges and new opportunities for its widespread use. Kong and Marawar address the knowledge gap about the often ignored, highest-risk for ASyS demographical segment—the older adults. Yoo reviews the current literature on BIRDs (Brief Potentially Ictal Rhythmic Discharges) that have a high degree of association with ASyS and status epilepticus and subsequent outcomes.

We will be remiss not to point out the lack of articles that can guide us on ASyS and EA management in the collection. However, it merely reflects the malaise toward ASyS management research in the neurological community. A phenomenal boost to help overcome this apathy would be to define etiology-specific ASyS using multimodal biomarkers, which will be a big step toward improving management, prognosis, and understanding epileptogenesis.

## Author contributions

VP, MG, ZC, and CB contributed to the conception of the editorial. VP wrote the first draft of the manuscript. All authors contributed to the manuscript revision, read, and approved the submitted version.
